# Relationship between maternal adverse childhood experiences and infant development: A systematic review (protocol)

**DOI:** 10.1097/MD.0000000000014644

**Published:** 2019-03-08

**Authors:** Renata de Barros Bruno Ximenes, José Christian Machado Ximenes, Simony Lira Nascimento, Sarah M. Roddy, Álvaro Jorge Madeiro Leite

**Affiliations:** a Master's Student of Federal University of Ceará and Professor of Unichristus – Christus University Center; b Neurologist of Fortaleza General Hospital; c Assistant Professor of Federal University of Ceará, Fortaleza, Ceará, Brazil; d Associate Dean of Loma Linda University, Loma Linda, CA; e Pediatrician and Full Professor of Medical School of Federal University of Ceará, Fortaleza, Ceará, Brazil.

**Keywords:** ACEs, adverse childhood experiences, child development, maternal adverse childhood experiences, systematic review protocol

## Abstract

**Introduction:**

Twenty years ago, the first study was conducted to access adverse childhood experiences (ACEs) and their relation to outcomes in adulthood. The effects of exposure to childhood trauma can also be transmitted to other generations. There are some studies that suggest the hypothesis that intergenerational transmission may begin during intrauterine life through the change in placental-fetal physiology due to maternal exposure to adverse events in childhood. Those exposures can lead to a variety of conditions such as altered brain architecture, increase in placental corticotrophin hormone (pCRH) at the end of gestation, or emotional and behavioral changes during childhood and adolescence. The systematic review, therefore, is established to determine if there is a reliable association between maternal ACEs in childhood and altered child development.

**Method:**

We will conduct a systematic review according to the guidelines of the meta-analysis of observational studies in epidemiology (MOOSE) and with the preferred reporting items for systematic review with a focus on health equity (PRISMA-E). A comprehensive search strategy will be conducted in the following databases: MEDLINE, EMBASE, CINAHL, Web of Science, SCOPUS, Lilacs, and SciELO. Following a 2-step screening process, data including the full reference, objectives, target population, description of the exposure (ACEs), outcome measures, study design, length of follow-up period, and the study results will be extracted, synthesized, and reported. Risk of bias and quality of the studies will also be assessed.

**Dissemination and ethics:**

The results of this review will be disseminated through peer-reviewed publication. Because all of the data used in this systematic review has been published, this review does not require ethical approval.

**Discussion:**

This systematic review of the last 20 years will summarize and present the evidence for the relationship between maternal ACEs and the development of her child.

**Systematic Review registration:**

PROSPERO #CRD42018111456.

## 1 Introduction

Twenty years ago, Felitti and colleagues^[1]^ conducted the first study to access adverse childhood experiences (ACEs) and their relation to outcomes in adulthood. ACEs are sources of stress that people may experience early in life, usually before age 18. They are recognized as a public health problem, which can affect the health and well-being of children, not only at the time when ACEs occur but also later in life.^[1,2]^ Such experiences include multiple types of abuse (physical, sexual, and psychological), neglect, and various types of family dysfunction.^[3]^ In addition to having psychological consequences, several studies have shown that ACEs are associated with health-related risk factors such as substance abuse, risky sexual behavior, obesity, cardiovascular disease, cancer, and diabetes.^[1,2,4,5]^ Having multiple ACEs is an important risk factor for several unfavorable health outcomes. The results suggest that the impact of these adverse experiences in childhood on adult health status is strong and cumulative.^[6]^

Early childhood severe stress produces a cascade of events that has the potential to alter brain development. These changes, in turn, increase the risk of developing post-traumatic stress disorder (PTSD), depression, symptoms of attention deficit hyperactivity disorder, borderline personality disorder, dissociative identity disorder, and substance abuse.^[3,5–8]^ The risk factors assessed in the ACE Study include examples of several stressors that are capable of inducing a toxic stress response. Toxic stress is a strong, frequent, or prolonged activation of the body's stress response systems (cortisol hormone) in the absence of an adult supportive relationship. It leads to disruption of brain circuits, altering brain architecture in a phase of high brain plasticity when the brain is highly sensitive to chemical changes.^[3]^ Epigenetic factors (especially deoxyribonucleic acid [DNA] methylation) may also occur as a signature of these environmental experiences in the genome, without altering the nucleotide sequence, thus affecting long-term phenotypes.^[3,9,10]^ These changes may occur in the prenatal period, after birth or during childhood.^[11,12]^

The effects of exposure to childhood trauma can also be transmitted to other generations.^[13]^ The same authors suggest the hypothesis that intergenerational transmission may begin during intrauterine life through the change in placental-fetal physiology due to maternal exposure to adverse events in childhood, specifically in the release of placental corticotrophin hormone (pCRH). Pregnant women who suffered childhood trauma showed a 25% increase in pCRH at the end of gestation compared to women not exposed to ACEs. Increased maternal and fetal cortisol concentrations in pregnancy were associated with changes in children's temperament and behavior,^[14,15]^ delay in cognitive development^[6,8,16]^ and changes in the regulation of the hypothalamic-pituitary-adrenal axis.^[17]^

In a recent study, Moog and colleagues^[18]^ examined the intergenerational effect of maternal exposure to childhood traumas on the offspring's brain structure versus the control group. The difference observed in the brain's volume of offspring of mothers exposed to ACEs was 6% less than brain's volume of the offspring of non-exposed mothers, especially in the cortical gray matter. In another paper by the same group, a model is proposed that suggests that intrauterine life represents a particularly sensitive period regarding the effects of maternal exposure to ACEs, and its effects can be transmitted to the offspring.^[19]^

In a systematic review, there was evidence of a global association between the history of maltreatment in the mother's childhood and her child's emotional and behavioral changes during childhood and adolescence. Maternal psychological crisis and poor parental practices were the main means of mediating this association.^[20]^

To the extent that abuse and other potentially harmful experiences in childhood contribute to the development of these risk factors, such childhood exposures should be recognized as underlying causes of adult morbidity and mortality.^[8]^

The purpose of this systematic review is to synthesize the empirical literature of the past 20 years on the relationship between the history of adverse events in maternal childhood and the development of their children. The systematic review, therefore, is established to determine if there is a reliable association between maternal ACEs in childhood and altered child development.

## 2 Methods

### 2.1 Protocol and registration

We will perform a systematic review to identify published articles on the impact of adverse experiences in maternal childhood on the development of their offspring. The review will be completed and reported according to the guidelines of the meta-analysis of observational studies in epidemiology (MOOSE)^[21]^ and with the preferred reporting items for systematic review with a focus on health equity (PRISMA-E).^[22]^ This SR is also registered with PROSPERO (registration # CRD42018111456).

### 2.2 Eligibility criteria and results of interest

To identify relevant studies, specific inclusion and exclusion criteria have been identified using the population, exposure, controls, outcomes, and study designs (PECOS)^[22]^ criteria as follows: population: mothers who suffered adverse experience in the first 18 years of life and the development of their children and compare them with children of mothers who did not suffer adverse experience in childhood. Exposure: maternal ACEs. Controls: mothers who were not exposed to ACEs and their offspring. Outcomes: the negative impact on child development (global or/and cognitive or/and language or/and socioemotional/behavioral) or/and brain architecture. Study designs: observational method study (i.e., cohort, cross-sectional, case–control) examining the link between maternal adverse childhood experience and its impact on child health and development.

#### 2.2.1 Primary outcomes

(1) Infant global development.

#### 2.2.2 Secondary outcomes

(1) Cognitive;
(2) Language;
(3) Socioemotional/behavioral;
(4) Brain architecture.

### 2.3 Search strategy

Studies will be searched on electronic databases from 1998 onwards and published in English, Spanish or Portuguese: MEDLINE (via PubMed) EMBASE, SciELO, LILACS, Scopus, Web of Science, and CINAHL. The literature searches of peer-reviewed publications will be supplemented by scanning the reference lists of relevant studies and systematic reviews. The main search strategy will be based on the following terms and will be adapted to the requirements of the databases: (“maternal adverse child∗ experience∗” or “maternal child∗ abuse” or “maternal child∗ maltreatment ”or “maternal child∗ trauma” or “maternal child∗ victim∗” or “maternal child∗ neglect”) and (“Child development”). Supplementary searches of key journal and of gray literature websites will be undertaken. All results will be imported into citation management software, where duplicate citations will be screened and removed.

### 2.4 Screening

We will use a 2-step process to assess the results of the literature search. We developed screening questions based on the inclusion/exclusion criteria for both screening levels. Before conducting the formal screening, a calibration exercise will be undertaken to pilot test and refine our screening questions. At first, titles and abstracts retrieved from the database and web searches and citations of relevant studies will be screened by 2 independent researchers to assess their potential relevance for full review. Any discrepancies will be resolved through discussion with a third reviewer if indicated. The second step, 2 reviewers will independently assess the full text of all retained records. Discrepancies will be resolved by consensus or by a third reviewer. As the publication objectives indicate, the following kind of studies will be included: observational method study (i.e., cohort, cross-sectional, case–control) examining the link between maternal adverse childhood experience and its impact in child health and development. Inclusion criteria:

(1) published in English, Spanish, or Portuguese
(2) included measurement of maternal maltreatment experiences in the childhood period (<18 years),
(3) measured child development (global or/and cognitive or/and language or/and socioemotional/behavioral) or/and brain architecture in the childhood period (<18 years) and
(4) tested for association between (2) and (3). Exclusion criteria: studies that evaluated child development in an indirect way (evaluating maternal and socioeconomic health indicators).

### 2.5 Data extraction

Before performing data extraction, a calibration exercise will be undertaken to pilot test and refine our data extraction form. Two reviewers will independently extract and document data from each included study. All abstracts and titles of retrieved citations will be screened for eligibility. Articles will be retrieved for full-text review if they met inclusion criteria. Extracted information will include: general study characteristics (title and authors, year of study, geographical location); study characteristics: study design and method of data-analysis; participants: study population, number of participants in each group, patient characteristics such as age, gender, co-morbidities; child health outcome (as reflected in primary and secondary outcomes) and the presence of adverse events in the mother's childhood accessed through questionnaire. For studies with insufficient data to evaluate the eligibility, we will contact the study authors by email at least twice for clarification. The study will be excluded if there is still insufficient data following this process.

### 2.6 Assessing risk of bias/quality of the evidence

Quality assessments will be undertaken by 2 independents reviewers. Any discrepancies will be resolved through discussion with a third reviewer. Discordant results were resolved by consensus following the PECOS steps (patient, exposure, controls, outcomes, and study) being used for final inclusion in the study. The methodological quality of each study will be evaluated using the strengthening the reporting of observational studies in epidemiology scale (STROBE).^[23]^ The initiative STROBE developed a checklist of 22 items, the STROBE Statement, with recommendations about what should be included in a more accurate and complete description of observational studies. A table with details of risk of bias in individual studies will be provided. We will use the grading of recommendations assessment, development, and evaluation (GRADE) framework to assess the quality of the body of evidence.^[24]^

### 2.7 Analysis/synthesis

Data will be entered into Microsoft Office Excel with the following topics: author and year, study design, characteristics of the sample, instrument used to do access maternal ACEs, and to identify/trial child development, as the secondary outcomes. Results from the studies will be summarized and tabulated according to the variables listed above and discuss data in a narrative review. We intend to use Software R for statistical analysis. If sufficient data is available from the included studies, subgroup analyses will be conducted to compare the effect of exposure on different population subgroups (e.g., different ages) and/or different ACEs exposure types (e.g., physical abuse, neglect, etc) and/ or different outcomes (primary or secondary). Where statistical pooling is not possible, the findings will be presented in narrative form. However, we are unable to specify the subgroups in advance.

### 2.8 Quality assurance

The proposed SR will be reported according to the guidelines of the MOOSE^[21]^ and PRISMA format.^[22,25]^ The MOOSE checklist resulting from workgroup deliberations is organized around recommendations for reporting background, search strategy, methods, results, discussion, and conclusions. The PRISMA consists of a checklist of 27 essential items for transparent reporting of SRs.

## 3 Discussion

This systematic review will summarize and present the evidence base for the relationship between maternal ACEs and the development of her child for the last 20 years since the first ACEs study was done. This review will also inform further research and will lay the groundwork for more empirical studies on this theme. These results may also be important for policymakers, decision-makers, clinicians, and patients/families who are involved in the health care system.

## Author contributions

**Conceptualization:** Renata B. B. Ximenes; Jose Christian M. Ximenes; Álvaro J. M. Leite.

**Data curation:** Renata B. B. Ximenes; Jose Christian M. Ximenes.

**Formal analysis:** Renata B. B. Ximenes; Jose Christian M. Ximenes; Álvaro J. M. Leite.

**Investigation:** Renata B. B. Ximenes.

**Methodology:** Renata B. B. Ximenes; Simony L. Nascimento; Álvaro J. M. Leite.

**Project administration:** Renata B. B. Ximenes; Álvaro J. M. Leite.

**Resources:** Renata B. B. Ximenes; Sarah M. Roddy.

**Supervision:** Sarah M. Roddy; Álvaro J. M. Leite.

**Validation:** Renata B. B. Ximenes.

**Visualization:** Renata B. B. Ximenes.

**Writing – original draft:** Renata B. B. Ximenes; Jose Christian M. Ximenes; Sarah M. Roddy.

**Writing – review & editing:** Renata B. B. Ximenes; Jose Christian M. Ximenes; Simony L. Nascimento; Sarah M. Roddy; Álvaro J. M. Leite.

Renata de Barros Bruno Ximenes orcid: 0000-0002-2511-5336.
